# Prise en charge du cancer du col utérin durant la grossesse: à propos de 05 cas

**DOI:** 10.11604/pamj.2014.19.245.5094

**Published:** 2014-11-05

**Authors:** Abderrahman El Mazghi, Touria Bouhafa, Kaoutar Loukili, Hanan El Kacemi, Issam Lalya, Khalid Hassouni

**Affiliations:** 1 Faculté de Médecine, Université Sidi Mohamed Ben Abdallah, Service de Radiothérapie, CHU Hassan II, Fès, Maroc; 2 Service de Radiothérapie, Institut National d'Oncologie, Rabat, Maroc; 3 Service de Radiothérapie, HIM Mohamed V, Rabat, Maroc

**Keywords:** Col utérin, cancer, grossesse, cervical cancer, cancer, pregnancy

## Abstract

L'association d'un cancer du col utérin avec une grossesse est une éventualité rare. Son incidence est assez mal évaluée, elle se situe selon les études entre 1 et 2/10 000. Lorsque la découverte en est faite, il faut conjuguer deux impératifs parfois totalement divergents: le traitement de la mère et la prise en charge du foetus. Cette association pose schématiquement quatre grands problèmes, qui sont: Celui du diagnostic, qui est loin d’être évident, compte tenu des remaniements cervicaux observés en début de gestation, le pronostic de l'affection, la date du traitement chirurgical et du délai entre le diagnostic et la prise en charge thérapeutique, enfin et de manière plus accessoire, le devenir de la grossesse et le mode d'accouchement. Nous rapportons une série de 5 cas de cancer du col utérin découverts au cours de la grossesse colligés dans notre service entre 2010 et 2013. La prise en charge thérapeutique est identique à celle des patientes en dehors de la grossesse même si quelques adaptations sont nécessaires du fait de l’état gravide, le pronostic du cancer ne semble pas être modifié par la grossesse.

## Introduction

Le diagnostic du cancer invasif du col utérin (CCU), évoluant au cours d′une grossesse n′est pas toujours évident. L'incidence au cours de la grossesse des dysplasies est estimée à 1% et celle du cancer du col utérin à un sur 10 000. Ce dernier est l'un des cancers les plus diagnostiqués durant la grossesse avec le cancer du sein, avec les lymphomes et les mélanomes. Toutefois, l'incidence exacte demeure imprécise [1]. Pour les patientes peu suivies, le début de grossesse doit être l'occasion de réaliser un frottis s'il n'a pas été fait récemment et de sensibiliser les patientes à la nécessité de cette surveillance [2]. La recommandation d'un frottis systématique en début de grossesse va augmenter l'incidence de découverte de lésions précancéreuses, d'où l'importance de définir des conduites à tenir claires. Pour les cancers survenant pendant la grossesse, les objectifs sont doubles et parfois antagonistes: réaliser une prise en charge plus proche de celle des patientes non enceintes, c'est-à-dire ne pas sous-traiter à cause de la grossesse et si possible, conserver la grossesse. Si auparavant, une interruption de grossesse était souvent recommandée au cours des deux premiers trimestres, des publications récentes rapportent des cas de préservation de la grossesse. Cela ne doit pas être au détriment du résultat carcinologique [3].

## Méthodes

Notre étude est une analyse rétrospective portant sur 5 cas de carcinome épidermoide du col utérin au cours de la grossesse colligés aux services de gynécologie et de radiothérapie des CHU Hassan II de Fès et d'Avicenne de Rabat du 01 janvier 2010 au 31 décembre 2013. Notre analyse s'est penchée sur les caractéristiques cliniques, anatomopathologiques et thérapeutiques.

## Résultats

L′âge médian de nos parturientes était de 38 ans avec des extrêmes de 35 et 42 ans et l’âge de la grossesse au moment du diagnostic était de 28 semaines avec des extrêmes de 10 et 37 semaines. Les symptômes initiaux les plus fréquents étaient les métrorragies (Tableau 1). La taille moyenne des lésions était de 3 cm de diamètre. L′examen anatomopathologique a montré un carcinome épidermoide dans tous les cas. La maladie a été classée selon la classification FIGO 2009. Le traitement consistait en une interruption médicale de la grossesse ou une césarienne, complétée par une chirurgie et/ou une radiothérapie plus au moins une chimiothérapie concomitante en fonction de l’âge gestationnel et du stade tumoral. Aucune récidive locale, locorégionale ou générale n′a été notée avec un recul moyen de 36 mois (Tableau 2).

**Tableau 1:** Caractéristiques cliniques des parturientes

Cas clinique (année)	Age (ans)	Age gestationnel (SA)	Circonstance de diagnostic
1(2010)	42	20	Métrorragies
2(2010)	38	10	Découverte fortuite
3(2011)	40	28	Métrorragies
4(2012)	35	30	Découverte fortuite
5(2013)	38	37	Métrorragies

Durant la période de 2010à 2013, 5 cas de carcinomes épidermoide pendant la grossesse ont été colligés aux services de gynécologie et de radiothérapie

**Tableau 2:** Caractéristiques anatomopathologiques et thérapeutiques

Cas clinique	Taille tumorale (cm)	FIGO	Histologie	Délai diagnostic/traitement	Traitement
1	5	IB2	CE peu différencié	2 semaines	IMG+RCC
2	1.5	IB1	CE peu différencié	1 semaine	IMG+CHEL+RTH
3	2	IB1	CE peu différencié	6 semaines	Ces à 34 SA +CHEL+RTH
4	2	IB1	CE peu différencié	4 semaines	Ces à 34 SA +CHEL+RTH
5	4.5	IB2	CE peu différencié	1 semaine	Ces à 37 SA +RCC

**FIGO**: Fédération internationale de gynéco-obstétrique, RTH: radiothérapie externe, CE: carcinome épidermoïde. **Ces**: Césarienne, IMG: interruption médicale de la grossesse, CHEL: hystérectomie élargie +lymphadenectomie, **SA**: semaine d'aménorrhée, RCC: radio-chimiothérapie concomitante

## Discussion

Le diagnostic du CCU pendant la grossesse est le plus souvent réalisé sur frottis. Pendant la grossesse, bien qu'il existe une tendance à la surestimation lésionnelle du fait des modifications stromales et des glandes endométriales, le taux de frottis ASC (atypies des cellules malpighiennes) est plus élevé, par ailleurs, la sensibilité du frottis est la même qu'en dehors de la grossesse [4, 5]. En cas d'anomalies cytologiques évoquant une lésion de haut grade et frottis ASC-H (lésion malpighienne intra-épithéliale de haut grade) ou un carcinome invasif, une colposcopie avec biopsie doit être rapidement réalisée. La colposcopie demeure l'examen de référence en cas d'anomalies cytologiques pendant la grossesse. Les signes d'infiltration sont les mêmes que ceux reconnus en dehors de la grossesse, principalement l'ulcération, la vascularisation atypique et une zone acidophile très épaisse. Ces lésions nécessitent de réaliser une biopsie. Même s'il existe un risque d'hémorragie plus important, celle-ci peut être contrôlée par tamponnement, voire électrocoagulation ou suture et ce risque ne doit pas remettre en cause la biopsie si celle-ci apparaît nécessaire [4, 6].

Les métrorragies sont le principal signe clinique. Elles ne doivent pas être à tort imputées à la grossesse. Devant toute métrorragie de la grossesse, l'examen doit débuter par une pose de spéculum pour identifier l'origine du saignement. Le bilan d'extension de la tumeur repose sur l'examen clinique et l'IRM abdominopelvienne (classiquement sans injection de gadolinium au premier trimestre). Une radiographie de thorax peut être réalisée (avec protection foetale) après le premier trimestre pour des tumeurs localement avancées (≥4 cm) [5]. La prise en charge thérapeutique dépend du stade (et de la taille tumorale), du type histologique de la tumeur, du terme de la grossesse et du désir du couple à conserver éventuellement la grossesse. Les facteurs pronostiques sont les mêmes qu'en dehors de la grossesse, les deux principaux étant le stade et l'atteinte ganglionnaire. La discussion doit impliquer gynécologues, oncologues médicaux, radiothérapeutes, radiologues et pathologistes pour définir une prise en charge carcinologique « optimale » mais aussi obstétriciens et néonatologistes pour obtenir le meilleur compromis entre le pronostic maternel et le pronostic foetal. Si, dans la prise en charge de la patiente, un accouchement est envisagé avant 38 SA, celui-ci devrait être réalisé dans un centre périnatal dont le niveau est adapté au terme de l'accouchement [7]. Après la maturité foetale (34-35 SA), il est possible de préserver la grossesse tout en ne prenant pas de retard dans la prise en charge thérapeutique de la lésion cervicale qui sera réalisée, selon les standards, après l'accouchement. La date de l'accouchement sera déterminé en fonction du terme au moment du diagnostic et du degré d'urgence à traiter la lésion cervicale (taille et stade tumoral). L'accouchement doit être réalisé par césarienne. Avant maturité foetale, s'il s'agit d'une tumeur de type histologique « classique » et que la patiente souhaite préserver sa grossesse, le traitement va dépendre du stade de la maladie et du terme. En effet, la possibilité de réaliser certains gestes de stadification dépend du volume utérin: jusqu’à 20-24 SA, la lymphadénectomie laparoscopique pelvienne première est le plus souvent réalisable sans être trop gêné par le volume utérin. Elle permet d'identifier le sous-groupe de patientes qui a le plus mauvais pronostic, c'est-à-dire avec atteinte ganglionnaire et dont le traitement ne peut être différé après l'accouchement [4–7].

Pour les tumeurs de stade précoce avec un curage pelvien négatif, on proposera une surveillance pendant la grossesse sans traitement de la lésion cervicale. Cette surveillance sera clinique et radiologique (IRM abdominopelvienne toutes les quatre à huit semaines bien qu'il n'y ait pas de consensus concernant la fréquence de surveillance). En absence de progression tumorale, la tumeur cervicale sera traitée dès que la maturité foetale sera atteinte. L'accouchement sera réalisé par césarienne et le traitement de la lésion cervicale sera réalisé selon les standards. Si le curage pelvien est positif, l'interruption médicale de grossesse doit être proposée et le traitement doit être une radio-chimiothérapie concomitante première (après obtention de la vacuité utérine). Le niveau d'extension des champs de la radiothérapie dépendra du niveau de l'extension ganglionnaire (pelvienne seule ou pelvienne et lombo-aortique). L'atteinte lombo-aortique pourra être précisée, soit par une lymphadénectomie lombo-aortique laparoscopique, soit par un PET scanner réalisé après l'expulsion du foetus [7].

Pour les tumeurs de stade avancé (à partir du stade IB2 selon la classification de la FIGO), le traitement standard est la radio-chimiothérapie concomitante. Si la tumeur est diagnostiquée avant 22 à 24 SA, une interruption de grossesse est recommandée. Après 24 SA (sous réserve de l'absence d'extension radiologique ganglionnaire et extra-pelvienne), la radiochimiothérapie peut débuter après la césarienne qui sera faite dès que la maturité foetale le permet (à condition que l'obtention de celle-ci ne retarde pas la mise en route du traitement de la tumeur de plus de six à huit semaines) [8]. Une option peut se discuter chez des patientes souhaitant préserver leur grossesse: la chimiothérapie néoadjuvante. Ce traitement ne peut être proposé que chez des patientes ayant un terme de grossesse supérieur à 20 SA et en ayant prévenu celles-ci des risques carcinologiques éventuels, décrits dans la littérature, d’échec du traitement et donc, de progression tumorale avec mise en jeu du pronostic vital et de l'incertitude sur les conséquences foetales de la chimiothérapie [9]

Dans notre série, deux patientes ont bénéficié d'une interruption de la grossesse, la première parturiente parce qu'elle avait une tumeur localement avancé (stade IB2) et la deuxième parce qu'elle était en début de grossesse (10SA). Une chirurgie radicale a été faite chez elles suivie d'une radio chimiothérapie ou radiothérapie seule selon le cas. La césarienne avec Colpohysterectomie élargie avec lympadenéctomie ont été réalisé dés la maturité foetale à 34 SA chez deux patientes suivies d'une radiothérapie. Le pronostic de l′affection est loin d′être une simple question théorique. Il conditionne en effet la date de mise en route du traitement. Un début de réponse peut être apporté pour les stades précoces de la maladie (Ia, Ib et IIa); l′étude de Van der Vange portant sur 44 patientes atteintes d′un cancer du col appariées à une population contrôle de femmes atteintes non enceintes mais présentant les mêmes caractéristiques d′âge, de grade, de stade, de type tumoral et de modalités de prise en charge thérapeutique, ne montre pas de différences statistiques de survie pour ce qui concerne les stades précoces. Compte tenu de la faiblesse de l′échantillon, aucune constatation ne peut être effectuée pour les stades avancés [10]. Hopkins, dans les mêmes conditions ne retrouve pas non plus de différence de survie, en appariant sa population de 35 patientes enceintes de stade IB et 170 patientes de même âge moyen, non enceintes, traitées de 1960 à 1989. La période de découverte ne semble pas modifier le pronostic [11]. La Survie à 5 ans était de 90% pour 10 patientes pour lesquelles la découverte du CCU s′est effectuée au second trimestre, 75% (5 patientes) au troisième trimestre et 75% pour le post-partum (20 patientes) [12, 13].

## Conclusion

Chez les parturientes peu suivies ou en l'absence de dépistage depuis plus de deux ans, un frottis doit être réalisé en début de grossesse pour dépister des anomalies cervicales et sensibiliser les patientes à l'intérêt de ce dépistage. Pour les lésions de dysplasie en l'absence d'invasion prouvée à la colposcopie, le traitement peut être différé au post-partum, sous couvert d'une surveillance rapprochée. Pour les lésions invasives, le bilan doit être complété par IRM pour définir au mieux la taille de la lésion. La prise en charge dépendra du terme, du stade de la lésion et de l'atteinte ganglionnaire, si cette information peut être obtenue (lymphadénectomie pelvienne par voie coelioscopique jusqu’à 20-24 SA pour les tumeurs de moins de 4 cm). Le couple doit être informé que la grossesse en elle-même ne modifie pas le pronostic de la tumeur. Si le couple choisit de conserver la grossesse et que le traitement doit être différé pour attendre la maturité foetale, les risques liés au délai doivent être évalués en fonction du délai nécessaire et des facteurs pronostiques de la tumeur. Le traitement est décidé de manière collective entre oncologue, chirurgien-oncologue, obstétricien, néonatologistes et le couple.
